# Rapid Urban Malaria Appraisal (RUMA) IV: Epidemiology of urban malaria in Cotonou (Benin)

**DOI:** 10.1186/1475-2875-5-45

**Published:** 2006-06-02

**Authors:** Shr-Jie Wang, Christian Lengeler, Thomas A Smith, Penelope Vounatsou, Martin Akogbeto, Marcel Tanner

**Affiliations:** 1 Swiss Tropical Institute (STI), P.O. Box, CH-4002 Basel, Switzerland; 2 Centre de Recherche Entomologique de Cotonou (CREC), Ministère de la Santé Publique, B. P. 06-2604, Cotonou, Benin

## Abstract

**Background:**

An estimated 40 % of the population in Benin lives in urban areas. The purpose of the study was to estimate malaria endemicity and the fraction of malaria-attributable fevers in health facilities in Cotonou.

**Methods:**

A health care system evaluation and a series of school parasitaemia surveys and health facility-based surveys were carried out during the dry season in of 2003, applying standard Rapid Urban Malaria Appraisal (RUMA) methodology. This study was part of a multi-site assessment supported by the Roll Back Malaria Partnership.

**Results:**

The field work was carried out in February-March 2003. In 2002 and out of 289,342 consultations in the public health facilities of Cotonou there were 100,257 reported simple malaria cases (34.6%) and 12,195 complicated malaria cases (4.2%). In the school parasitaemia surveys, a malaria infection was found in 5.2 % of all samples. The prevalence rates of parasitaemia in the centre, intermediate and periphery zones were 2.6%, 9.0% and 2.5%, respectively. In the health facility surveys the malaria infection rates in presenting fever cases were 0% (under one year old), 6.8% (one to five years old), 0% (> five to 15 years old) and 0.9% (over 15 years old), while these rates in the control group were 1.4%, 2.8%, 1.3% and 2.0%. The malaria-attributable fractions among presenting fever cases were 0.04 in the one to five years old and zero in the three other age groups. Hence, malaria played only a small role in fever episodes at the end of the dry season. In total, 69.2% of patients used a mosquito net the night before the survey and 35.1% used an insecticide-treated net, which was shown to be protective for an infection (OR = 0.23, 95% CI 0.07–0.78). Travelling to a rural area (5.8% of all respondents) did not increase the infection risk.

**Conclusion:**

The homogenously low malaria prevalence might be associated with urban transformation and/or a high bednet usage. Over-diagnosis of malaria and over-treatment with antimalarials was found to be a serious problem.

## Introduction

Heterogeneity in urban malaria transmission patterns is driven mostly by human activity, urban development and environmental determinants. The dynamics of urbanization in sub-Saharan Africa (SSA) greatly affect eco-systems and, hence, population health and this is currently insufficiently documented at present [1-4].

In 2000 it was estimated that 40.1 % of the population in Benin lived in urban areas, and the country can be considered urbanized by SSA standard. The climate in Cotonou is characterized by two rainy seasons and two dry seasons. A major dry season starts in December and ends at the month of March. This is followed by a major rainy season from April to the end of July. A minor dry season starts in August and extends to the middle of September, followed by a minor rainy season to the end of November. One of the most serious environmental problems in Cotonou is the abundance of water, especially during the two rainy seasons. Over half of Cotonou suffers every year from several months of flooding, allowing mosquito larvae breeding and leading to an increase in malaria transmission. The variability of malaria transmission and vector densities in Cotonou and their association with lagoon salinity have been well described [5-10].

In June, 2002 a standard protocol for a Rapid Urban Malaria Appraisal (RUMA) was developed on the basis of a WHO proposal and an Environmental Health Project draft protocol [11,12]. The present work was carried out as a part of a multi-site assessment in three francophone countries (Côte d'Ivoire, Burkina Faso and Benin) and one anglophone country (Tanzania). This standard RUMA included: a literature review, the collection of available health statistics, a series of school parasitaemia surveys, a series of health facility surveys, malaria risk mapping and a brief review of the country's health care system. At each of the four sites the RUMA aimed to provide an overview of the urbanization history, parasite rates for different zones, an estimate of the fraction of malaria-attributable fevers, an outline of health care services and highlights of the "lessons learned". This paper is the fourth in a series of four country assessment papers. A separate overview considers this work in a wider context [13].

## Methods

### Study sites and sample selection

Cotonou is situated in the smallest department of Benin, Littoral. Cotonou, as a whole, counts as one "commune" (urban district), consisting of six administrative zones (Cotonou One to Six) and 138 sub-zones. It is the largest city and main port of Benin, situated between latitude 6.2°-6.3° N and longitude 2.2°-2.3° E. It was built on a sandy beach to create a harbour close to the only waterway between the Gulf of Guinea and Lake Nokoué. The waterway divides Cotonou into two parts: Cotonou One to Three are located on its eastern side and Cotonou Four to Six are located on its western side. The total area is 74 square kilometre. The population has multiplied rapidly from 3,300 inhabitants in 1921 to 383,000 in 1981 and 780,657 in 2002. In order to implement the RUMA, the territory of Cotonou was divided into three different areas (centre, intermediate and periphery), based on population density, distance to the centre and environmental characteristics, as defined by a survey of Akobeto *et al*. [6]. The coastal commercial area is considered as "centre"; the lakeside areas are considered as "periphery" and in between are the "intermediate/residential" areas. While this classification bears no relationship with the six administrative zones, it is congruent with the known malaria transmission patterns in Cotonou [6] and hence relevant for our aim to briefly assess infection rates and their variability in the city. In each of these three areas one health facility and one school were selected for the surveys.

#### Centre

Centre de Santé Saint Michel and Gbéto primary school are at the geographical centre of Cotonou, near the new bridge crossing the inlet channel, where the biggest market of Benin is located. The catchments areas of the Centre de Santé Saint Michel are Sikecodji, Gbénomey, Maro-militaire and St. Jean to the south of Cotonou Five, bordering with Cotonou Three. These areas encompass the organised business districts.

#### Intermediate zone

Hôpital Saint Luc and Sainte-Rita primary school are located in the north of Cotonou Five, which is affected by flooding. Hôpital Saint Luc is the biggest hospital in Benin under catholic administration. In 1996, 64% of attendees at Hôpital St Luc were residents of Cotonou Five. Residents from the catchments area of Centre de Santé Saint Michel, in particular Sikecodji, Gbénomey, Saint Michel, Maro-militaire or Saint Jean also visited Hôpital Saint Luc.

#### Periphery

Centre Médical de Ménontin and Ménontin primary school are situated on the north-west department border near the town of Godomey. This area is frequently affected by floods. The patients came not only from the Ménontin community but also from other peripheral areas of Cotonou and neighbouring Abomey-Calavi, at the west end of Cotonou. During recent years the World Bank has implemented an urban upgrade plan in the Ménontin, including a low-income household water-pipe connection programme, emergency road and drainage operations, as well as the construction of the Centre Médical de Ménontin [14]. By contrast, the intermediate zones surrounding the Hôpital Saint Luc have many unplanned settlements and are underserved.

### RUMA methodology

#### Review of literature and collection of health statistics

Published information on malaria epidemiology was reviewed systematically through a literature search in the main bibliographic databases (PUBMED and EMBASE), through scanning reference lists and through contacting relevant experts, nationally and internationally. The demographic and health system information, as well as routine malaria reports, were collected from the Ministry of Health of Benin (MOH), the Institut National de la Statistique et de l'Analyse Economique (INSAE) and USAID-Benin.

### School parasitaemia surveys

A cross-sectional parasitaemia survey was carried out in the three selected primary schools during March 2003. At the onset of the study we estimated that 5 % to 50% of fever cases among children under 15 years of age were due to malaria. Hence, a sample size of 200 in each facility allowed to get an estimate of the proportion of cases with parasites within plus/minus 5% for the whole range. Most children were aged 6–12 years, with only child aged five years. After obtaining informed consent from parents and teachers, thick and thin blood smears were collected from at least 200 school children in each school and stained with Giemsa. Both thin and thick blood slides were read at the Centre de Recherche Entomologique de Cotonou (CREC). The parasite density was defined as the number of parasites per 200 white blood cells. The children were interviewed with the assistance of school teachers regarding their family situation, malaria infection history and malaria prevention. Each of the selected schools was close to the health facility chosen for the fever survey. In total, 234, 296 and 204 children from Gbéto, Sainte-Rita and Ménontin primary schools were recruited. Numbers of children exceeded 200 in the three schools, especially in Sainte-Rita, so more children were recruited in the survey.

### Health facility fever surveys

From the end of March to April 21, 2003, a total of 200 fever cases and 200 non-fever controls were recruited from each of the 3 selected health facilities, with half the participants being under 5 years of age. The recruitment of participants was *ad hoc *and sequential over this time period and we applied standard inclusion and exclusion criteria. The inclusion criteria for cases were: outpatients with a history of fever (past 36 hours) or a measured temperature of ≥ 37.5°C. The controls were recruited from another department of the same clinic without current or past fever, matched by age and residency. Exclusion criteria were: patients with signs of severe disease, patients returning to the health facility for follow-up visits, non-permanent town residents (less than six months per year). About 50% of the sample was aged ≤5 years. After being recruited and giving informed consent, each patient had an axillary temperature measurement with an electronic thermometer and a blood film taken. Each participant or their guardian were then interviewed about socio-economic status and malaria treatment and prevention history (questionnaire similar to the one used for school surveys). An armpit temperature reading is usually 0.3°C to 0.6°C lower than an oral temperature reading and therefore 0.5°C was added to the digital readout, giving the final temperature used in the analysis. From these results odds ratios (OR) were calculated as the proportion of odds of having parasitaemia in fever cases over the odds of having parasitaemia in controls. Further, the fraction of fevers attributable to malaria was calculated using the formula: (1-1/Odds Ratio)*P, with P being the proportion of fever episodes with malaria parasites [15].

### Brief description of the health care system

Senior officers of the MOH and INSAE provided information about the structure of the government health care system, the reforms of health service delivery and the number of providers for malaria diagnosis and treatment in Cotonou.

### Mapping

Due to technical and manpower limitations, it was not possible to proceed with the mapping of health facilities and mosquito breeding sites, as proposed in the standard RUMA methodology. This part was actually found to be too demanding in terms of manpower and time to fit in the framework of a rapid assessment of six-10 weeks duration. This component could only be carried out in Ouagadougou and Dar es Salaam, where the support of outside expertise could be relied upon [13]. In any case, this component should not be part of a standardized basic RUMA.

### Statistical methods

The data were double-entered and validated in EpiInfo 6.04 (CDC Atlanta, USA, 2001). The data analysis was carried out in Stata 8 (Stata Corp. Texas, USA, 2003). The X^2 ^test was applied to assess associations between categorical variables. Logistic regression was performed to assess the association between binary outcomes and different explanatory variables.

### Ethics

In the absence of a national ethics committee, the study received clearance from the national institutional review board of the CREC. All the patients gave informed consent. A prescription of chloroquine or amodiaquine was paid for if the patients presented fever signs, or when they were found to be parasitaemic.

## Results

An outline of the main results is given below. A comprehensive report on the work in Cotonou is available from the authors.

### Brief description of the health care system

The public health system is divided administratively into three levels: central/national, departmental, and peripheral/community [16-18]. The MOH of Benin has been in the process of reorganizing its structure through the creation of an intermediate level in each department: health zones (in French: *zones sanitaires*) to facilitate decentralized planning and increase the efficiency of resource allocation. Each health zone authority is in charge of two levels: 1) regional hospitals (in French: *hôpitaux de zone*) at the intermediate level and 2) community health districts (in French: *circonscriptions sanitaires de commune*), which consist of health centres, dispensaries, maternity wards and village health units at the periphery level.

In November 2002, there were 34 public health facilities in Cotonou with only 368 beds for hospitalization (Table 1), serving a total population of 780,000 [19,20]. There were 331 private health services including many NGO/religious facilities. This is in line with the MOH's *Evaluation du Système National d'Information et de Gestion Sanitaire *(SNIGS) annual statistics report for 1999, which estimated that the private sector offered a significant amount (30%) of health services in Cotonou. The workload of public health services was found to be heavy, with one doctor/nurse serving more than 2,000 people and one laboratory serving at least 52,000 people (9,000 people for each laboratory technician). The provision of public and private health services is heterogeneous in Cotonou. Most of them are located in Cotonou Three and Six, near the business centre and the residencies of the upper class and foreigners.

**Table 1:** Public and private health services in Cotonou in 2002.

**Zone**	**Public health facilities**										**Total public** **health facilities**	**Total private** **health facilities** **(NGOs & private)**	**Total**
			
	CSSP/CSSCU	CCS		Other health facilities						UVS			
	Without operating theatres	MCH	Dispensary	MCH	Dispensary	TB control clinic	Leprosy centre	Nursing school	Labs	Open			
**Cotonou 1**	1	0	1	1	3	0	1	3	1	0	**11**	**24**	**35**
**Cotonou 2**	1	1	2	0	0	0	0	1	1	2	**8**	**47**	**55**
**Cotonou 3**	1	0	0	0	0	0	0	0	1	0	**2**	**78**	**80**
**Cotonou 4**	1	0	0	0	0	0	0	0	0	0	**1**	**39**	**40**
**Cotonou 5**	1	0	1	0	0	0	0	2	1	0	**5**	**68**	**73**
**Cotonou 6**	1	0	1	0	0	1	0	3	1	0	**7**	**75**	**82**

**Total**	**6**	**1**	**5**	**1**	**3**	**1**	**1**	**9**	**5**	**2**	**34**	**331**	**365**

**CCS : **Complexe Communal de Santé, **CSSP : **Centre de Santé de Sous-Préfecture, **CSSCU : **Centre de Santé de Circonscription Urbaine, **MCH : **Maternal and Child Health Clinic, **NGOs : **Non-Governmental Organisations, **UVS : **Unités Villageoises de Santé, **TB **: Tuberculosis

### Results of malaria routine reports

Malaria morbidity and mortality data in Cotonou are collected on an annual basis. The original datasets and monthly reports were not available at the INSAE, and their accuracy and completeness could, therefore, not be ascertained. The available reports summarized all the clinical diagnoses in public health facilities from 1996 to 2000, differentiating simple (mild) malaria and complicated (severe) malaria by age and sex. Unfortunately, the data were not broken down by month and so the seasonality could not be investigated. Hence, only the inter-annual and intra-city patterns could be observed. There was no information on how many health facilities reported regularly to INSAE and the municipal health department. Neither was the number of laboratory-confirmed malaria cases mentioned in the report.

**Figure 1:**
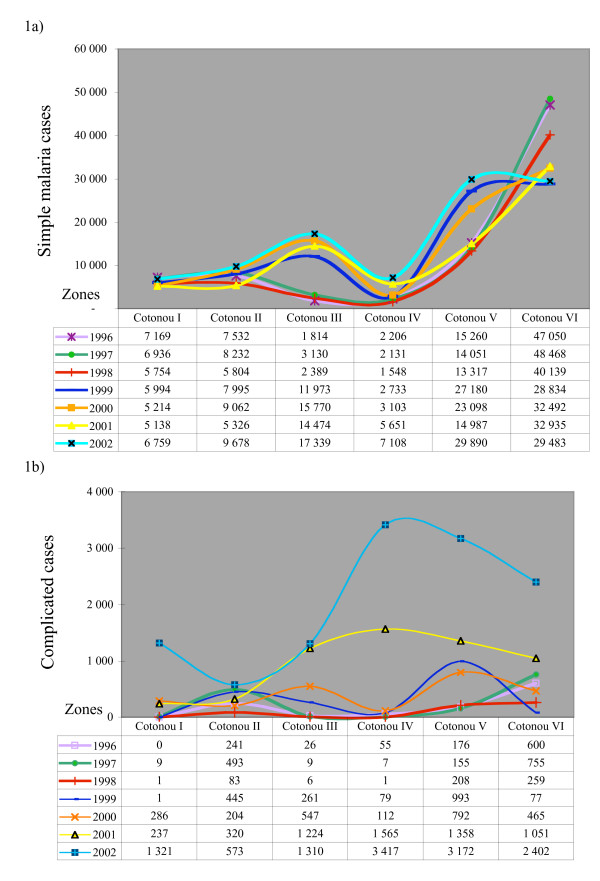
a) Reported mild (simple) malaria cases in Cotonou, 1996–2002. b) Reported severe (complicated) malaria cases in Cotonou, 1996–2002.

In 2002, there were 100,257 simple malaria cases (34.5% of total) and 12,195 complicated cases (4.2% of total) reported among 289,342 consultations in the public health facilities of Cotonou. Between 1996 and 2002, on average 34% of total clinical consultations were attributed to simple malaria and 1–4.2% to complicated malaria cases (Figure 1a &1b). In Cotonou One and Two, the annual total remained fairly stable during the past 6 years, while the reported malaria cases increased substantially in Cotonou Three, Four and Five. Unfortunately it is not possible to know whether these trends were real or linked to reporting differences. However, the downward trend in Cotonou Six could well be due to the fact that while it used to be a remote area it is now almost entirely occupied by a well-to-do community.

### School parasitaemia surveys

**Figure 2:**
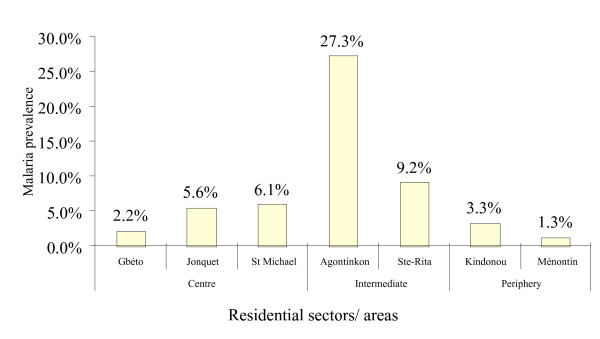
Distribution of malaria prevalence rates by different zones of Cotonou Five. School parasitaemia surveys. N = 734.

Malaria infections were found in 34 of 734 valid blood films (5.2 %, 95% CI: 0.04–0.07). The prevalence rates of parasitaemia in the centre, intermediate and periphery areas were 2.6% (95% CI: 0.01–0.05), 9.0% (95% CI: 0.06–0.13) and 2.5% (95% CI: 0.01–0.06), respectively. Parasitaemia was only found in seven sectors: 1) centre: Gbéto, Jonquet and St Michael, 2) intermediate: Agontinkon and Ste-Rita, 3) periphery: Kindonou and Ménontin (Figure 2). Overall, the prevalence rates were much lower than expected from previous reports.

### Health facility-based surveys

Among 1,288 blood films, 22 samples were detected with trophozoites and three with gametocytes of *Plasmodium falciparum *(1.9%). Only seven (1.8%) out of 386 fever cases and 18 (2.0%) out of 902 non-fever controls were positive. The mean parasite density in the positives was 2,729 trophozoites/μl (maximum: 25,600/μl). The majority of infected patients had low parasitaemia (1–400/μl) with only three having hyperparasitaemia (> 6,400/μl).

All samples were divided into four age groups: infants <one year old, children of one to five years, children > five to 15 years and adults (>15 years old). The overall prevalence rates of parasitaemia were 1.0%, 4.2%, 0.9% and 1.7% in these age groups. Malaria parasites were found in 0%, 6.8%, 0% and 0.9% of fever cases (same age categories) and in 1.4%, 2.8%, 1.3% and 2.0% of the control group (Table 2). The odds ratio for parasitaemia in fever cases ranged from 0 to 2.64. The fractions of malaria- attributable fevers could not be calculated using the standard formula for the infants and children aged six to 15 years (because of the division by zero), but since the cases did not have any parasites, the fractions were equalled to zero. Hence, the fractions of malaria-attributable fever were very low: 0, 0.04, 0 and -0.01 for the age categories above.

**Table 2:** Malaria prevalence in fever cases and controls and the fractions of malaria-attributable fevers by age group. Health facilities surveys. Na = not applicable (when the OR = 0 this value cannot be calculated using the standard formula).

Age group	Fever cases	Controls	OR	95% CI	Fractions of malaria attributable fevers
Infants 0–1 year	0/63 (0%)	2/140 (1.4%)	0.00	0.00–9.16	Na
Children 1–5 years	5/68 (6.8%)	4/137 (2.8%)	2.64	0.59–12.19	0.04
Children 6–15 years	0/35 (0%)	1/78 (1.3%)	0.00	0.00–40.03	na
Adults >15 years	2/213 (0.9%)	11/529 (2.0%)	0.45	0.10–2.05	-0.01^¥^

^¥ ^Since the OR<1, the fraction of malaria attributable fevers is <0, suggesting that the true rate of malaria fevers is zero.

**Figure 3:**
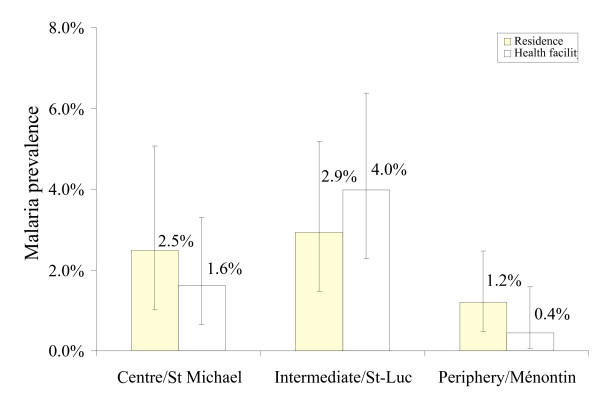
Malaria prevalence by residential areas and location of health facilities. Vertical bars represent 95% confidence intervals. Health facility-based surveys. N = 386 fever cases and 902 controls.

**Figure 4:**
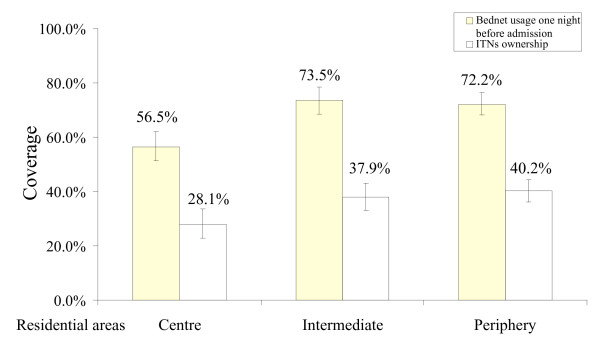
Use of bednets and insecticide-treated nets (ITNs) the night before interview, by residential areas. Vertical bars represent 95% confidence intervals. Health facility-based surveys.

The results of the health facility surveys were similar to the results of the school surveys: people living in the intermediate zone of Cotonou had a similar risk of malaria infection than those from the city centre and the periphery (Figure 3). The result of a logistic regression model adjusted for age groups was not significant for the risk gradient, in part because of the small number of malaria cases (intermediate area: OR = 1.19, 95% CI = 0.45–3.13, periphery: OR = 0.47, 95% CI = 0.16–1.36 compared to city centre). The bednet usage and ITN ownership rates were high in Cotonou: 69.2% and 35.1%, respectively (Figure 4). A logistic regression model adjusted for residential areas and age groups showed that all bednets seemed to reduce the malaria risk but this was not significant (OR = 0.61, 95% CI 0.26–1.40), while treated nets (ITNs) significantly reduced malaria infections (OR = 0.23, 95% CI = 0.07–0.78, P = 0.018).

The education level of residents and the source of water supply were homogeneous throughout Cotonou. More residents in the periphery areas lived near agriculture land or gardens. A majority of people lived in concrete/brick houses. After adjusting for different residential areas, the logistic regression analysis showed that there was a strong association between malaria infections and housing material: the OR of mud buildings was 6.2 compared to concrete/brick buildings (95% CI = 1.3–29.6, P < 0.05). However, the proportions of residents living in a concrete/brick building in the central, intermediate and peripheral areas of Cotonou were high (96.1%, 97.1% and 97.9%) and individuals living in mud houses are likely to have belonged to the lowest socio-economic group with an expected higher risk for malaria. Only 5.8% (N = 75) of the study population had travelled to rural areas within the last three months and none had malaria parasites in their blood.

Overall, 32% (N = 425) of the participants claimed having had malaria treatments one month prior to the survey. Among them, nearly 50% self-treated at home and 46.0 % were previously treated in health centres or in a hospital.

## Discussion and conclusion

This study brought to light a number of important and relevant pieces of information. But it is good to also appreciate the limitations of the current RUMA, which is a cross-sectional study performed during a single season. The present assessment was carried out during the dry season (February-March) and similar surveys need to be repeated during the rainy season. The frequency of malaria parasites in presenting fever cases are likely to be higher then. Unfortunately, the routine statistics were not disaggregated by month, and hence, seasonality of reported malaria cases could not be described.

The annual routine data showed that, on average, 34% of the consultations in health facilities had a diagnosis of "clinical malaria", and this fraction has been fairly constant over the last seven years. However, our results suggest that only a small fraction of these cases could really be attributed to malaria (0–4% depending on the age groups). A similar situation exists in the three other cities (Abidjan, Ouagadougou, Dar es Salaam) in which studies were carried out in the frame of the present multi-site RUMA [13]. In Cotonou, another survey was conducted in the paediatric ward of the main university hospital from April, 1988 to March, 1989 and the overall malaria infection rate was 20% among 480 hospitalized children aged 0–14 years [21]. In that study the malaria infection rate was around 15% in February and 5% in March and it increased to 30% in April. Another fever survey was carried out between April, 1994 and March, 1995, with 325 randomly selected households being visited weekly [22]. The results showed that city children had 0.3 febrile episodes annually. Both studies showed that in March (at the end of one of the two dry periods) malaria accounted for less than 10% of all consultations in public health facilities.

These findings clearly indicate that there is a high level of over-diagnosis of malaria in the dry season in Cotonou and a resulting massive over-treatment. The overall prevalence of parasitaemia among the febrile cases in health facilities was lower than 2%, while the routine reports of the previous year suggested that 34.2% of total consultations were due to malaria. Such a big discrepancy is a major public health issue and it needs to be confirmed in the high transmission season. In other settings RUMA were done during a low-transmission period in Dar es Salaam in 2003 and during a high-transmission period in Abidjan and Ouagadougou in 2002; all these studies showed a similar high proportion of over-diagnosis of malaria [13].

This massive over-treatment raises two important issues. Firstly, over-treatment with antimalarials leads to a waste of resources and exposes patients unnecessarily to the side-effects of antimalarials. Secondly, there is a high risk of missing another diagnosis because the health care provider will focus on malaria and might not further investigate an alternative diagnosis. Even more than for simple malaria the latter is an issue in the case of patients diagnosed with severe malaria, as shown by two recent studies in Tanzania [23,24]. Beyond medical considerations, over-diagnosis and over-treatment might affect disproportionately the poorer segment of the population [25] and lead to unnecessary expenses in an economically vulnerable group. In view of the introduction of the next generation of more expensive malaria therapies (artemisinins-based combination therapy – ACT) a coordinated action on the systematic diagnosis of malaria in all presenting fever cases is urgently required and should be planned from the onset. The use of Rapid Diagnostic Tests (RDT) offers a practical way forward and this needs to be considered rapidly.

Our results confirm the results from Akogbeto *et al *[26] that both untreated and treated nets protect individuals from malaria.

It was surprising that there was a slightly lower malaria prevalence rate in the periphery compared to the intermediate zone. This could be explained by the salinity of the lagoon and/or by the higher level of urbanization. The population density and the distribution of *Anopheles sp*. vectors is highly dependent on the salinity of the lagoon, with a higher proportion of *Anopheles melas *compared to the much more efficient vector *Anopheles gambiae s.l*. as salinity increases [6]. As a result, the level of malaria transmission was lower in communities near the beach (where standing water was much saltier) than in other areas: about five infected bites per person per year versus 29 infected bites per person in the centre of Cotonou. The higher risk of malaria infections may also have been associated with poor urban agriculture irrigation systems.

The findings of this study have also serious implications for the assessment of the burden of malaria in urban environments, since the routine statistics seriously inflate the true situation. Because of unspecific case definitions, poor documentation and deficiencies in reporting, the routine health data do not reflect the reality in most SSA cities. Many studies including the RUMA [13] have demonstrated that it is often impossible to rely on the routine data for assessing malaria endemicity and burden of disease.

Finally, RUMA conducted in four African cities allowed to give a good insight into urban malaria in SSA. Overall, there was a surprisingly low level of transmission heterogeneity in these cities, with the exception of Ouagadougou. The problem of over-diagnosis was common to all sites and this provided an important pointer for health planners. However, RUMA are too limited both in time and space to be useful on a larger scale. In the urban environment the malaria transmission situation is also likely to evolve rapidly in the future and this needs to be tracked. As a result, a more extensive and frequent surveying system is required. This should be achieved through the strengthening of routine data reporting and possibly through repeated surveys. In a second step, it is also important to carry out a more detailed risk mapping, so that specific control interventions such as larviciding or environmental management can be planned. However, such detailed surveys require substantially more time and resources [27] and they only make sense once additional resources for control become available. Cities are the demographic hubs of all SSA countries and generally their economic engines. These surveys suggest that malaria transmission is lower than previously thought in many cities and the high density of people per surface area make transmission control easier than in rural areas. This provides real opportunities that should be seized as rapidly as possible.

## Authors' contributions

SW participated in the design of the study, conducted the field work, analysed and interpreted data and drafted the manuscript. CL conceived the study, coordinated the field work and revised the manuscript. TS and PV assisted in the statistical analysis. MA supervised the data collection and laboratory work and commented on the manuscript. MT participated in the conception of the work, facilitated the overall coordination and revised critically the work at all stages.
